# Characteristics of recurrence, predictors for relapse and prognosis of rapid relapse triple-negative breast cancer

**DOI:** 10.3389/fonc.2023.1119611

**Published:** 2023-02-16

**Authors:** Shuang-Long Cai, Jing-Jing Liu, Ying-Xue Liu, Shao-Hong Yu, Xu Liu, Xiu-Quan Lin, Hong-Dan Chen, Xuan Fang, Tao Ma, Ya-Qing Li, Ying Li, Chun-Yan Li, Sheng Zhang, Xiao-Geng Chen, Xiao-Jing Guo, Jin Zhang

**Affiliations:** ^1^ The Third Department of Breast Cancer, Tianjin Medical University Cancer Institute and Hospital, National Clinical Research Center for Cancer, Tianjin, China; ^2^ Key Laboratory of Cancer Prevention and Therapy, Tianjin Medical University Cancer Institute and Hospital, Tianjin, China; ^3^ Tianjin’s Clinical Research Center for Cancer, Tianjin Medical University Cancer Institute and Hospital, Tianjin, China; ^4^ Key Laboratory of Breast Cancer Prevention and Therapy, Tianjin Medical University, Ministry of Education, Tianjin, China; ^5^ College of Basic Medical Sciences, Fujian Medical University, Fuzhou, China; ^6^ Department for Chronic and Noncommunicable Disease Control and Prevention, Fujian Provincial Center for Disease Control and Prevention, Fuzhou, China; ^7^ First Department of Cadre Clinic, Provincial Clinical Medical College of Fujian Medical University, Fujian Provincial Hospital, Fuzhou, China; ^8^ Department of Oncological Surgery, Provincial Clinical Medical College of Fujian Medical University, Fujian Provincial Hospital, Fuzhou, China; ^9^ Department of Breast Pathology and Lab, Tianjin Medical University Cancer Institute and Hospital, Tianjin, China

**Keywords:** triple negative breast cancer, rapid relapse, slow relapse, biological characteristics analysis, prognostic analysis

## Abstract

**Background:**

Triple-negative breast cancer (TNBC) patients who recur at different times are associated with distinct biological characteristics and prognoses. Research on rapid-relapse TNBC (RR-TNBC) is sparse. In this study, we aimed to describe the characteristics of recurrence, predictors for relapse, and prognosis in rrTNBC patients.

**Methods:**

Clinicopathological data of 1584 TNBC patients from 2014 to 2016 were retrospectively reviewed. The characteristics of recurrence were compared between patients with RR-TNBC and slow relapse TNBC(SR-TNBC). All TNBC patients were randomly divided into a training set and a validation set to find predictors for rapid relapse. The multivariate logistic regression model was used to analyze the data of the training set. C-index and brier score analysis for predicting rapid relapse in the validation set was used to evaluate the discrimination and accuracy of the multivariate logistic model. Prognostic measurements were analyzed in all TNBC patients.

**Results:**

Compared with SR-TNBC patients, RR-TNBC patients tended to have a higher T staging, N staging, TNM staging, and low expression of stromal tumor-infiltrating lymphocytes (sTILs). The recurring characteristics were prone to appear as distant metastasis at the first relapse. The first metastatic site was apt to visceral metastasis and less likely to have chest wall or regional lymph node metastasis. Six predictors (postmenopausal status, metaplastic breast cancer,≥pT3 staging,≥pN1 staging, sTIL intermediate/high expression, and Her2 [1+]) were used to construct the predictive model of rapid relapse in TNBC patients. The C-index and brier score in the validation set was 0.861 and 0.095, respectively. This suggested that the predictive model had high discrimination and accuracy. The prognostic data for all TNBC patients showed that RR-TNBC patients had the worst prognosis, followed by SR-TNBC patients.

**Conclusion:**

RR-TNBC patients were associated with unique biological characteristics and worse outcomes compared to non-RR-TNBC patients.

## 1 Introduction

Accounting for approximately 15%-20% of instances of breast cancer (BC), triple-negative breast cancer (TNBC) is pathologically defined by a lack of targetable estrogen receptor (ER), progesterone receptor (PR), and human epidermal growth factor receptor 2 (HER2) (1, 2). More than 35% of breast cancer-related deaths are caused by TNBC. Compared with other subtypes of BC, TNBC tends to have an increased aggressiveness, higher rate of metastasis, and shorter overall survival (OS) (3–5). The survival time of TNBC patients after the diagnosis of distant metastasis ranges from 17 to 25 months (6–8). Therefore, understanding the recurring characteristics, risk factors, and mechanisms of distant metastasis in TNBC patients are of critical importance to improving the prognosis of these patients.

Up to now, most studies on the recurring characteristics and risk factors for TNBC have focused on the overall recurring and metastatic populations, and have never further subdivided for all recurring and metastatic populations. To more accurately understand the differences in TNBC patients’ outcomes, we divided these patients into three groups: rapid relapse (RR-TNBC; distant relapse or death ≤2 years of diagnosis), slow relapse (SR-TNBC; distant relapse or death > 2 years), and no relapse (NR-TNBC;> 5 years no relapse/death) (9, 10). In several large TNBC cohort studies, the median time to distant metastasis was approximately 2 years, ranging from 19.7 to 31.2 months (6, 11–13). Therefore, we define RR-TNBC as distant relapse or death within 24 months of diagnosis. However, research on RR-TNBC is sparse. In this retrospective, single-institution study, we aimed to nvestigate the characteristics of recurrence, predictors for relapse, and prognosis in RR-TNBC patients.

## 2 Materials and methods

### 2.1 Patients

This retrospective analysis included 1584 consecutive triple-negative breast cancer (TNBC) patients pathologically confirmed in the Cancer Institute and Hospital of Tianjin Medical University from January 1, 2014, to December 31, 2016. The study protocol conformed to the ethical guidelines of the 1975 Declaration of Helsinki and was approved by the Medical Ethics Committee of Tianjin Medical University Cancer Institute and Hospital. Male patients, ductal carcinoma *in situ* (DCIS), lobular carcinoma *in situ* (LCIS), hormone receptor-positive (HR+) patients, human epidermal growth factor receptor 2 positives (HER2+) patients, patients with a history of other malignant tumors, bilateral breast cancer, or *de-novo* metastatic breast cancer patients were excluded. TNBC Patients with <2 years follow-up and no survival event were also excluded.

HER2 status was determined with immunohistochemistry (IHC) and/or fluorescence *in situ* hybridization (FISH) at the time of the first biopsy or breast surgery and classified according to the American Society of Clinical Oncology and the College of American Pathologists clinical practice guidelines for HER2 testing of 2013, respectively, and the Belgian Guidelines for HER2 testing (14). HER2 expression levels in TNBC included IHC 0, IHC 1+, or IHC 2+/FISH-negative. Hormone receptor and Ki67 status were determined by IHC using the Allred scoring system (15).

P53 expression ≤10% was considered P53-negative, P53 expression >10% was considered P53-positive.The expression levels of cytokeratin 5/6(CK5/6) and epidermal growth factor receptor (EGFR) <1% were considered to be negative, and positive if the expression levels of CK5/6 and EGFR were ≥1%. We evaluated stromal tumor-infiltrating lymphocytes (sTILs) on whole slides according to internationally established guidelines (16). sTILs were evaluated and grouped into three categories: low (≤10%), intermediate (10% to ≤40%), and high (>40%). All IHC readings were independently verified by two blinded and trained pathologists.

### 2.2 Follow-up strategy

Follow-up data of all TNBC patients in our study were gathered *via* telephone or our outpatient clinic, and the follow-up results were recorded. The follow-up time began with the first BC diagnosis. The follow-up deadline was June 1, 2022. Our follow-up strategies were as follows: routine breast/liver color ultrasound, ECT, chest and cranium CT plain scan for every initial TNBC patient to exclude the possibility of distant metastasis. In the first 3 years after surgery, we regularly reviewed breast/liver color ultrasound, X-ray, or chest CT plain scan every 3 months. Patients who survived 3-5 years after surgery were regularly reviewed with these items every 6 months. Patients who survived more than 5 years after surgery were regularly reviewed with these items every 1 year. Once distant metastasis was confirmed in follow-up patients, liver, lung, cranium CT, and ECT examinations were routinely performed at the same time.

### 2.3 Statistical analysis

Statistical analysis was performed using R version 4.1.0. The random seed was determined to be 123456. All TNBC patients were randomly divided into the training set and the validation set in a 7:3 ratio. The Chi-square test and Fisher’s exact test were used for comparison between the two groups. The multivariate logistic regression model was used to analyze the data of the training set. All variables in the univariate analysis with a P value <0.1 were included in a stepwise multivariable logistic regression model. The minimum AIC was used to determine the optimal logistic regression model. The C-index and calibration curve was used to evaluate the discrimination and accuracy of the multivariate logistic model. Kaplan-Meier (K-M) method and Log-rank test were used to evaluate the prognosis in all TNBC patients. The statistically significant difference was considered to be a P value<0.05.

## 3 Results

**Figure 1:**
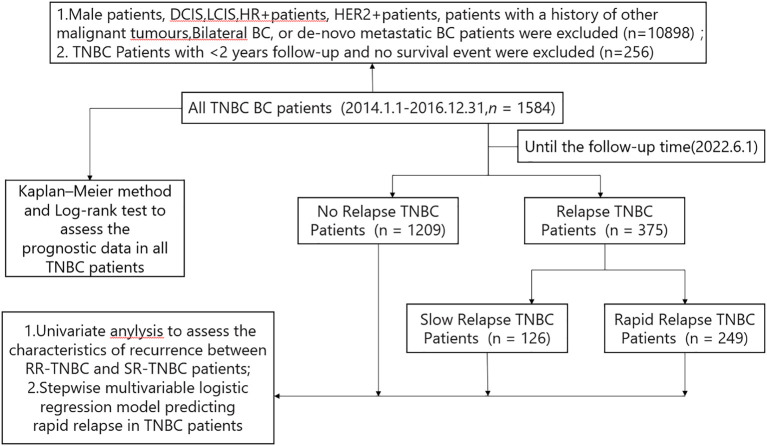
Constitution of the study population.BC, breast cancer; HR+,hormone receptor positive; HER2+, human epidermal growth factor receptor 2 positive; TNBC,triple negative breast cancer; RR-TNBC:rapid relapse-triple negative breast cancer; SR-TNBC:slow relapse-triple negative breast cancer; DCIS, ductal carcinoma in situ; LCIS, lobular carcinoma in situ.

A total of 12738 breast cancer patients from January 1, 2014, to December 31, 2016, were identified in the Cancer Institute and Hospital of Tianjin Medical University. Among these patients, 1584 TNBC patients (12.43%) fit the criteria to be included in the study. Among these 1584 TNBC patients,1209 patients had no relapse and 375 patients had relapse up until the time of follow-up data. Based on the time first distant metastasis occurred, 375 relapsed patients were divided into 249 patients with rapid-relapse(RR) and 126 patients with slow-relapse (SR) (**Figure 1**).

By comparing recurrence characteristics between 249 patients with RR-TNBC and 126 patients with SR-TNBC, RR-TNBC patients tended to have a higher T staging, N staging, TNM staging, and low expression of stromal tumor-infiltrating lymphocytes (sTIL). The recurring characteristics were prone to appear as distant metastasis at the first relapse. The first metastatic site was apt to visceral metastasis and less likely to have chest wall or regional lymph node metastasis (**Table 1**).

**Table 1:** The relapse and metastasis characteristics between RR-TNBC and SR-TNBC patients according to stratified variables: univariate analysis.

Variables	Rapid-relapse TNBC patients (n=249) No.(%)	Slow-relapse TNBC patients (n=126) No.(%)	P value
Age at diagnosis			0.142
≤35 years	30 (12.05)	9 (7.14)	
>35 years	219 (87.95)	117 (92.86)	
Menopausal status at diagnosis			0.099
Premenopausal	141 (56.63)	60 (47.62)	
Postmenopausal	108 (43.37)	66 (52.38)	
Family history			0.420
No	225 (90.36)	117 (92.86)	
Yes	24 (9.64)	9 (7.14)	
Type of surgery			0.853
Radical surgery	207 (83.13)	102 (80.95)	
Breast conserving surgery	36 (14.46)	21 (16.67)	
Lumpectomy surgery	6 (2.41)	3 (2.38)	
Pathological pattern			0.908
Invasive ductal carcinoma	210 (84.34)	108 (85.71)	
Metaplastic	18 (7.23)	9 (7.14)	
Others	21 (8.43)	9 (7.14)	
Tumour grade			0.064
G1/G2	99 (39.76)	51 (40.48)	
G3	150 (60.24)	72 (57.14)	
Missing	0 (0.00)	3 (2.38)	
Lymph-vascular invasion			0.731
No	108 (43.37)	57 (45.24)	
Yes	141 (56.63)	69 (54.76)	
Stromal tumor-infiltrating lymphocytes(sTIL)			<0.001
Low	123 (49.40)	36 (28.57)	
Intermediate	105 (42.17)	66 (52.38)	
High	21 (8.43)	24 (19.05)	
Tumor size staging			<0.001
pT0/Tis	6 (2.41)	0 (0.00)	
pT1	60 (24.10)	63 (50.00)	
pT2	132 (53.01)	57 (45.24)	
pT3	6 (4.76)	6 (4.76)	
pT4	12 (4.82)	0 (0.00)	
Nodal staging			<0.001
pN0	60 (24.10)	69 (54.76)	
pN1	81 (32.53)	45 (35.71)	
pN2	51 (20.48)	9 (7.14)	
pN3	57 (22.89)	3 (2.38)	
TNM staging			<0.001
0/I	15 (6.02)	30 (23.81)	
II	123 (49.40)	81 (64.29)	
III	111 (44.58)	15 (11.90)	
HER2 expression levels			0.606
0	90 (36.14)	39 (30.95)	
1+	99 (39.76)	54 (42.86)	
2+/FISH negative	60 (24.10)	33 (26.19)	
Ki-67			0.194
≤20	21 (8.43)	6 (4.76)	
>20	228 (91.57)	120 (95.24)	
P53			0.393
Negative (≤10)	78 (31.33)	45 (35.71)	
Positive (>10)	171 (68.67)	81 (64.29)	
CK5/6			0.480
Negative	57 (22.89)	33 (26.19)	
Positive	192 (77.11)	93 (73.81)	
EGFR			0.056
Negative	42 (16.87)	12 (9.52)	
Positive	207 (83.13)	114 (90.48)	
Type of chemotherapy			0.994
Anthracyclines	15 (6.02)	7 (5.56)	
Taxanes	9 (3.61)	4 (3.17)	
Anthracyclines+taxanes	207 (83.13)	105 (83.33)	
Combined with platinum	12 (4.82)	6 (4.76)	
None	6 (2.41)	4 (3.17)	
Adjuvant radiotherapy			0.434
No	168 (67.47)	90 (71.43)	
Yes	81 (32.53)	36 (28.57)	
First relapse forms			<0.001
L/R relapse at first	48 (19.28)	75 (59.52)	
Only DM at first	90 (36.14)	27 (21.43)	
DM and L/R relapse	111 (44.58)	24 (19.05)	
First metastatic site			
Visceral metastasis			<0.001
No	69 (27.71)	81 (64.29)	
Yes	180 (72.29)	45 (35.71)	
Brain metastasis			0.487
No	238 (95.58)	123 (97.62)	
Yes	11 (4.42)	3 (2.38)	
Bone metastasis			0.099
No	189 (75.90)	105 (83.33)	
Yes	60 (24.10)	21 (16.67)	
Metastatic site in the chest wall or regional lymph nodes			0.004
No	90 (36.14)	27 (21.43)	
Yes	159 (63.86)	99 (78.57)	

Family history: HBOC related cancer history; CK5/6: cytokeratin 5/6.

FISH, fluorescence in situ hybridization; EGFR, epidermal growth factor receptor.

L/R relapse at first: local/regional lymph nodes relapse at first.

Only DM at first: only distant metastasis at first.

DM and L/R relapse: distant metastasis and local/regional lymph nodes relapse.

R version 4.1.0 was used to randomly divide all 1584 TNBC patients into two groups in a 7:3 ratio: the training set (1108 patients) and the validation set (476 patients). The random seed was determined to be 123456. The clinicopathological parameters of these two groups are presented in **Table 2**. There was no statistical difference between these two groups in terms of clinicopathological parameters (**Table 2**). Through comparing the clinicopathological parameters between 175 RR-TNBC patients and 933 non-RR-TNBC patients in the training set, the univariate analysis showed that menopausal status, pathological pattern, T staging, N staging, TNM staging, sTIL expression levels, and HER2 expression levels were significantly associated with the risk factors for relapse in RR-TNBC patients (**Table 3**). Variables with P<0.1 in univariate analysis were included in multivariate analysis and the multivariate analysis showed that metaplastic breast cancer, ≥pT3 staging, and ≥pN1 staging were independent risk factors for relapse in RR-TNBC patients. Postmenopausal status, sTIL intermediate/high expression, and Her2 (1+) were independent protective factors for relapse in RR-TNBC patients (**Table 4**).

**Table 2:** Clinicopathological parameters of TNBC patients in the training and validation sets.

Variables	Training sets TNBC patients (n=1108) No.(%)	Validation sets TNBC patients (n=476) N o.(%)	P value
Age at diagnosis			0.064
≤35 years	79 (7.13)	47 (9.87)	
> 35 years	1029 (92.87)	429 (90.13)	
Menopausal status at diagnosis			0.880
Premenopausal	526 (47.47)	224 (47.06)	
Postmenopausal	582 (52.53)	252 (52.94)	
Family history			0.448
No	1003 (90.52)	425 (89.29)	
Yes	105 (9.48)	51 (10.71)	
Type of surgery			0.626
Radical surgery	928 (83.75)	401 (84.24)	
Breast conserving surgery	150 (13.54)	66 (13.87)	
Lumpectomy surgery	30 (2.71)	9 (1.89)	
Pathological pattern			0.438
Invasive ductal carcinoma	979 (88.36)	431 (90.55)	
Metaplastic	33 (2.98)	12 (2.52)	
Others	96 (8.66)	33 (6.93)	
Tumour grade			0.386
G1/G2	431 (38.90)	202 (42.44)	
G3	672 (60.65)	273 (57.35)	
Missing	5 (0.45)	1 (0.21)	
Lymph-vascular invasion			0.228
No	523 (47.20)	209 (43.91)	
Yes	585 (52.80)	267 (56.09)	
Stromal tumor-infiltrating lymphocytes(sTIL)			0.056
Low	311 (28.07)	106 (22.27)	
Intermediate	568 (51.26)	263 (55.25)	
High	229 (20.67)	107 (22.48)	
Tumor size staging			0.758
pT0/Tis	65 (5.87)	34 (7.14)	
pT1	485 (43.77)	202 (42.44)	
pT2	488 (44.04)	214 (44.96)	
pT3	58 (5.23)	20 (4.20)	
pT4	12 (1.08)	6 (1.26)	
Nodal staging			0.918
pN0	706 (63.72)	308 (64.71)	
pN1	238 (21.48)	104 (21.85)	
pN2	99 (8.94)	39 (8.19)	
pN3	65 (5.87)	25 (5.25)	
TNM staging			0.847
0/I	378 (34.12)	168 (35.29)	
II	559 (50.45)	239 (50.21)	
III	171 (15.43)	69 (14.50)	
HER2 expression levels			0.131
0	325 (29.33)	134 (28.15)	
1+	565 (50.99)	227 (47.69)	
2+/FISH negative	218 (19.68)	115 (24.16)	
Ki-67			0.575
≤20	104 (9.39)	49 (10.29)	
>20	1004 (90.61)	427 (89.71)	
P53			0.716
Negative	406 (36.64)	179 (37.61)	
Positive	702 (63.36)	297 (62.39)	
CK5/6			0.820
Negative	283 (25.54)	119 (25.00)	
Positive	825 (74.46)	357 (75.00)	
EGFR			0.808
Negative	180 (16.25)	75 (15.76)	
Positive	928 (83.75)	401 (84.24)	
Type of chemotherapy			0.821
Anthracyclines	58 (5.23)	20 (4.20)	
Taxanes	55 (4.96)	20 (4.20)	
Anthracyclines+taxanes	932 (84.12)	409 (85.92)	
Combined with platinum	41 (3.70)	16 (3.36)	
None	22 (1.99)	11 (2.31)	
Adjuvant radiotherapy			0.301
No	715 (64.53)	320 (67.23)	
Yes	393 (35.47)	156 (32.77)	

Family history, HBOC related cancer history;

CK5/6: cytokeratin 5/6;HER2:human epidermal growth factor receptor 2;

FISH, fluorescence in situ hybridization; EGFR, epidermal growth factor receptor.

**Table 3:** Risk factors of rapid relapse in training set according to stratified variables: univariate analysis.

Variables	Rapid-relapse TNBC patients (n=175) No.(%)	Non Rapid-relapse TNBC patients (n=933) No.(%)	P value
Age at diagnosis			0.077
≤35 years	18 (10.29)	61 (6.54)	
>35 years	157 (89.71)	872 (93.46)	
Menopausal status at diagnosis			0.005
Premenopausal	100 (57.14)	426 (45.66)	
Postmenopausal	75 (42.86)	507 (54.34)	
Family history			0.656
No	160 (91.43)	843 (90.35)	
Yes	15 (8.57)	90 (9.65)	
Type of surgery			0.808
Radical surgery	146 (83.43)	782 (83.82)	
Breast conserving surgery	23 (13.14)	127 (13.61)	
Lumpectomy surgery	6 (3.43)	24 (2.57)	
Pathological pattern			<0.001
Invasive ductal carcinoma	143 (81.71)	836 (89.60)	
Metaplastic	14 (8.00)	19 (2.04)	
Others	18 (10.29)	78 (8.36)	
Tumour grade			0.809
G1/G2	66 (37.71)	365 (39.12)	
G3	109 (62.29)	563 (60.34)	
Missing	0 (0.00)	5 (0.54)	
Lymph-vascular invasion			0.948
No	83 (47.43)	440 (47.16)	
Yes	92 (52.57)	493 (52.84)	
Stromal tumor-infiltrating lymphocytes(sTIL)			<0.001
Low	89 (50.86)	222 (23.79)	
Intermediate	69 (39.43)	499 (53.48)	
High	17 (9.71)	212 (22.72)	
Tumour size staging			<0.001
pT0/Tis	3 (1.71)	62 (6.65)	
pT1	46 (26.29)	439 (47.05)	
pT2	93 (53.14)	395 (42.34)	
pT3	25 (14.29)	33 (3.54)	
pT4	8 (4.57)	4 (0.43)	
Nodal staging			<0.001
pN0	43 (24.57)	663 (71.06)	
pN1	59 (33.71)	179 (19.19)	
pN2	33 (18.86)	66 (7.07)	
pN3	40 (22.86)	25 (2.68)	
TNM staging			<0.001
0/I	11 (6.29)	367 (39.34)	
II	90 (51.43)	469 (50.27)	
III	74 (42.29)	97 (10.40)	
HER2 expression levels			0.003
0	68 (38.86)	257 (27.55)	
1+	70 (40.00)	495 (53.05)	
2+/FISH negative	37 (21.14)	181 (19.40)	
Ki-67			0.211
≤20	12 (6.86)	92 (9.86)	
>20	163 (93.14)	841 (90.14)	
P53			0.119
Negative	55 (31.43)	351 (37.62)	
Positive	120 (68.57)	582 (62.38)	
CK5/6			0.375
Negative	40 (22.86)	243 (26.05)	
Positive	135 (77.14)	690 (73.95)	
EGFR			0.587
Negative	26 (14.86)	154 (16.51)	
Positive	149 (85.14)	779 (83.49)	
Type of chemotherapy			0.991
Anthracyclines	10 (5.71)	48 (5.14)	
Taxanes	8 (4.57)	47 (5.04)	
Anthracyclines+taxanes	148 (84.57)	784 (84.03)	
Combined with platinum	6 (3.43)	35 (3.75)	
None	3 (1.71)	19 (2.04)	
Adjuvant radiotherapy			0.118
No	122 (69.71)	593 (63.56)	
Yes	53 (30.29)	340 (36.44)	

Non-relapse TNBC patients include slow relapse and no relapse TNBC patients;

Family history,HBOC related cancer history;

CK5/6: cytokeratin 5/6;HER2:human epidermal growth factor receptor 2;

FISH, fluorescence in situ hybridization; EGFR, epidermal growth factor receptor.

**Table 4:** Multivariate Analysis for risk factors of rapid relapse in the training set.

Variable	Estimate	Se	Wald	p	OR(95%CI)	
Menopausal status at diagnosis						
Premenopausal	ref				
Postmenopausal	-1.069	0.207	26.666	<0.001	0.343 (0.229, 0.515)
Pathological pattern						
Invasive ductal carcinoma	ref				
Metaplastic	1.930	0.409	22.317	<0.001	6.889 (3.093, 15.343)
Others	0.009	0.354	0.001	0.981	1.009 (0.504, 2.017)
Stromal tumor-infiltrating lymphocytes(sTIL)						
Low	ref				
Intermediate	-0.606	0.224	7.295	0.007	0.546 (0.351, 0.847)
High	-1.181	0.359	10.821	0.001	0.307 (0.152, 0.621)
Tumor size staging						
pT0/Tis	ref				
pT1	0.332	0.672	0.244	0.621	1.394 (0.373, 5.201)
pT2	1.087	0.670	2.637	0.104	2.966 (0.798, 11.021)
pT3	1.498	0.745	4.039	0.044	4.473 (1.038, 19.277)
pT4	2.214	0.947	5.465	0.019	9.154 (1.430, 58.585)
Nodal staging						
pN0	ref				
pN1	1.775	0.234	57.533	<0.001	5.901 (3.730, 9.336)
pN2	1.936	0.306	39.959	<0.001	6.932 (3.803, 12.634)
pN3	3.204	0.386	68.794	<0.001	24.634 (11.553, 52.526)
Her2 expression levels						
0	ref				
1+	-0.537	0.221	5.891	0.015	0.585 (0.379, 0.902)
2+/FISH(-)	-0.309	0.281	1.202	0.273	0.734 (0.423, 1.275)

All variables in the univariate analysis with a P value <0.1 were included in the multivariate analysis.

**Figure 2:**
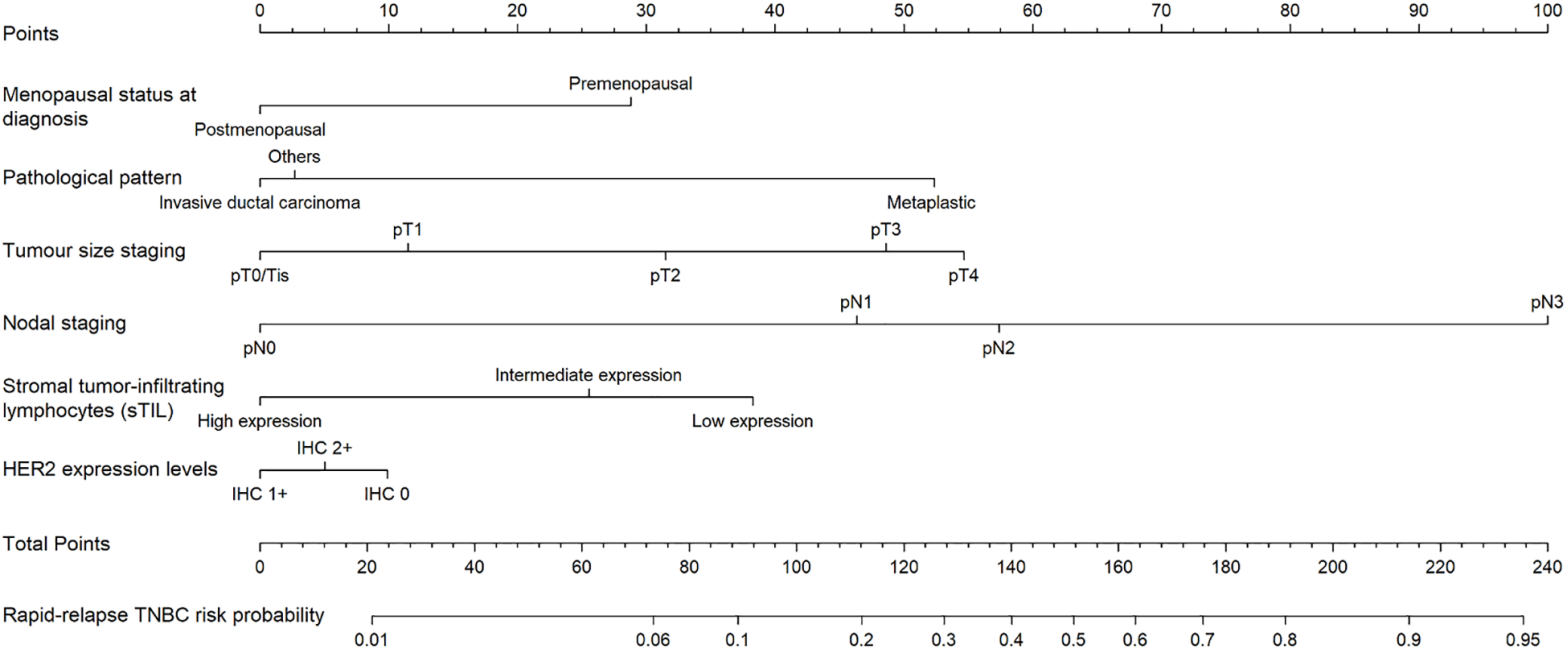
Nomogram for predicting rapid relapse of TNBC patients in the training set.

**Figure 3:**
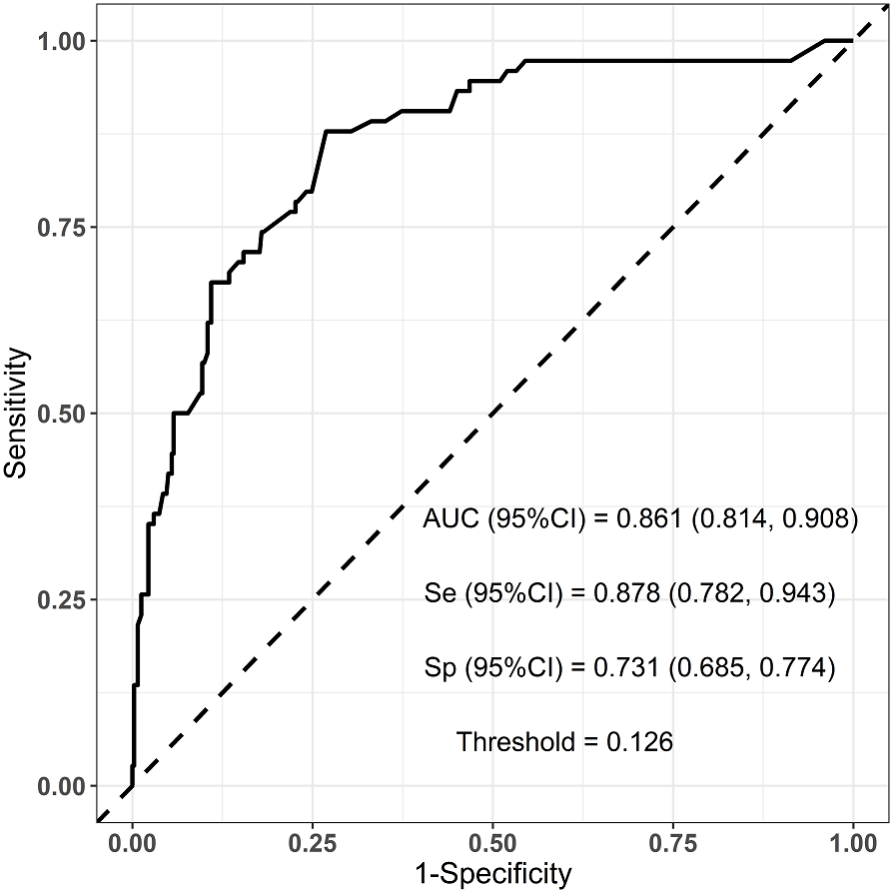
ROC analysis for predicting rapid relapse in the validation set. ROC, receiver operating characteristic; AUC, area under the curve.

**Figure 4:**
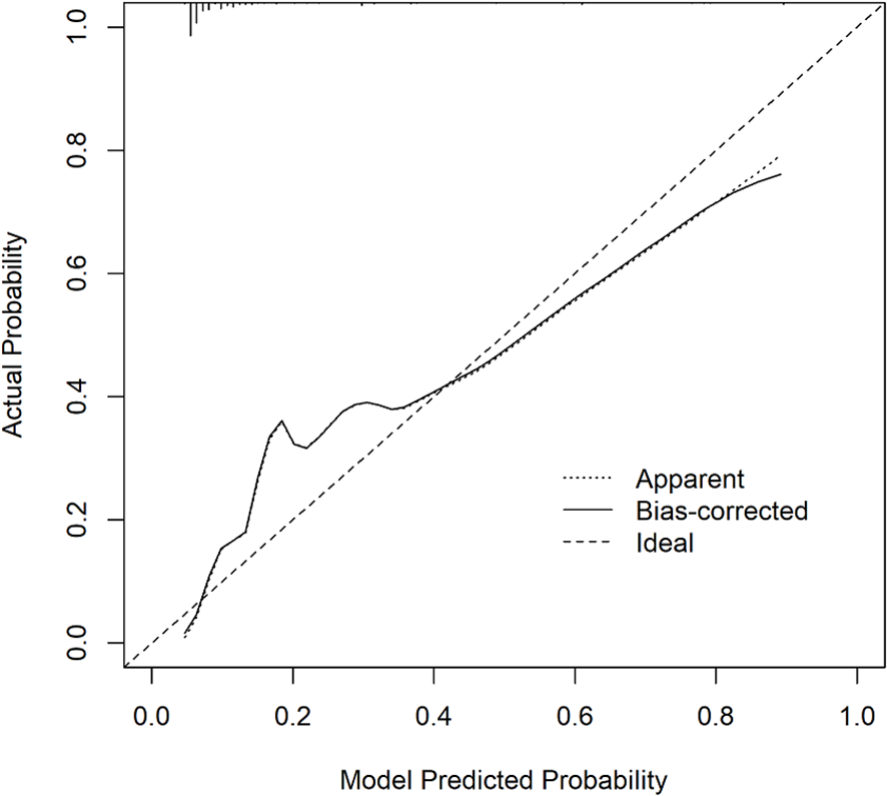
Calibration curve analysis for predicting rapid relapse in the validation set.

HER2, human epidermal growth factor receptor 2;

FISH(-), fluorescence in situ hybridization negative;

Six predictors (postmenopausal status, metaplastic breast cancer,≥pT3 staging,≥pN1 staging,sTIL intermediate/high expression, and Her2 [1+]) were used to construct the predictive model of rapid relapse in TNBC patients (**Figure 2**). The C-index and brier score in the validation set was 0.861 and 0.095, respectively. ROC (receiver operating characteristic) analysis for predicting rapid relapse in the validation set showed that AUC (area under the curve) was 0.861(95% confidence interval [CI]: 0.814–0.908). The sensitivity for predicting rapid relapse in the validation set was 0.878 (95% CI: 0.782–0.943). The specificity for predicting rapid relapse in the validation set was 0.731 (95% CI: 0.685–0.774) (**Figure 3**). The calibration curve for predicting rapid relapse in the validation set is presented in **Figure 4**. This suggested that the predictive model had high discrimination and accuracy.

**Figure 5:**
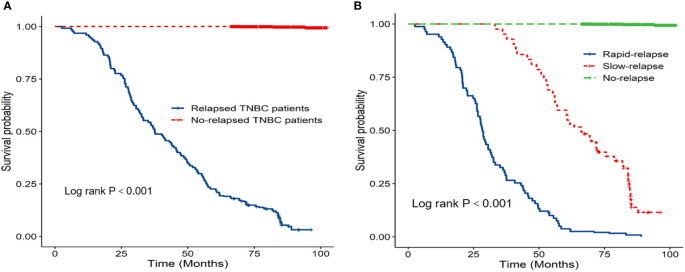
**(A)** K-M overall survival analysis of relapsed patients and no relapsed patients; **(B)** K-M overall survival analysis of different relapsed types in 1584 TNBC patients.

**Figure 6:**
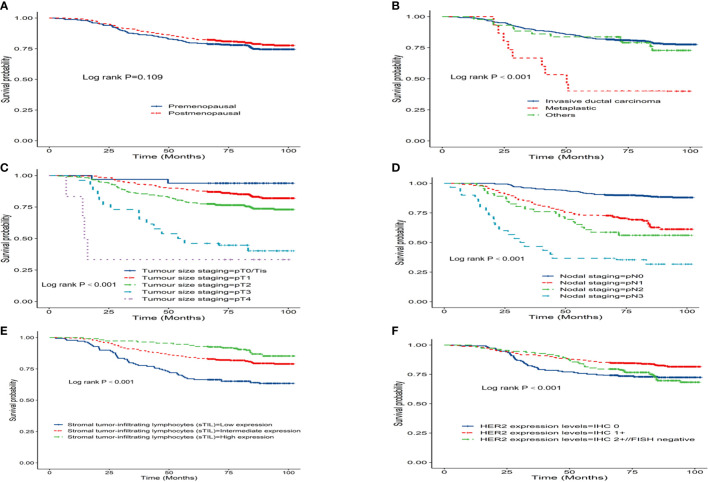
K-M overall survival analysis of all TNBC patients in six predictors for rapid relapse **(A)** Menopausal status at diagnosis; **(B)** Pathological pattern; **(C)** Tumour size staging; **(D)** Nodal staging; **(E)** Stromal tumor-infiltrating lymphocytes(sTIL); **(F)** Her2 expression levels).

**Figure 7:**
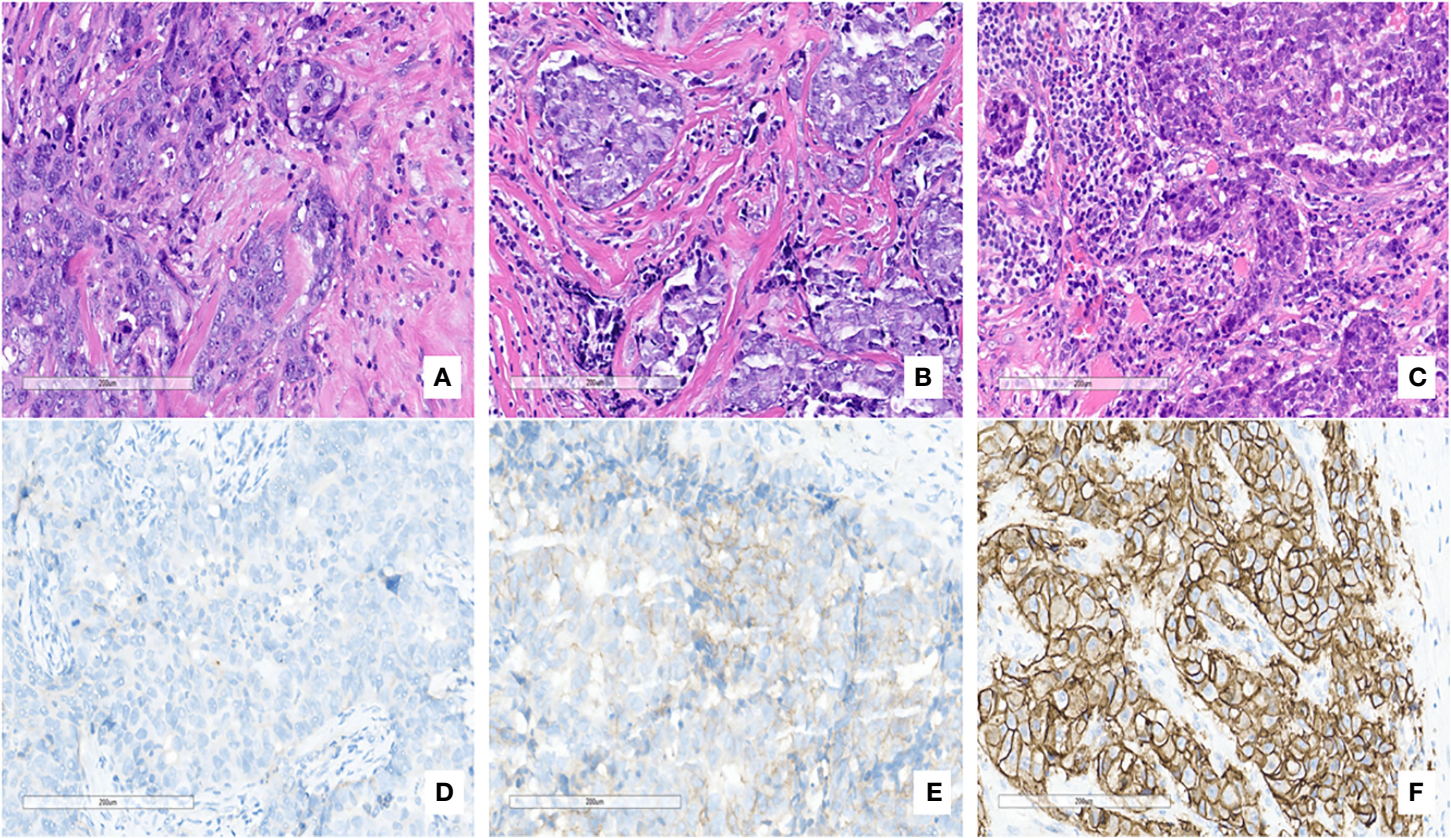
Different expressions of sTILs [**(A)** Low, **(B)** intermediate, **(C)** high] and different expression levels of Her2 [**(D)** IHC 0, **(E)** IHC 1+, **(F)** IHC 2+/FISH-negative] were detected in tumors by using hematoxylin and eosin staining (H&E×200).

Prognostic data of all TNBC patients showed that RR-TNBC patients had the worst prognosis, followed by SR-TNBC patients (**Figure 5**). The median disease-free survival and median overall survival of different recurred TNBC patients are presented in **Table 5**. The overall survival analysis of all TNBC patients in six predictors for rapid relapse are presented in **Figure 6**. Different expressions of sTILs(Low, intermediate, and high) and different expression levels of Her2 (IHC 0, IHC 1+, or IHC 2+/FISH-negative) were detected in tumors by using hematoxylin and eosin staining (H&E×200, **Figure 7**).

**Table 5:** Prognostic data in different relapsed TNBC patients.

Variables	All-relapse TNBC patients (n=125)	Rapid-relapse TNBC patients (n=83)	Slow-relapse TNBC patients (n=42)	P value
mDFS	16.9 (15.5, 18.5)	14.6 (13.7, 15.1)	42.1 (36.3, 45.8)	<0.001
mOS	37.6 (35.1, 43.8)	28.4 (27.5, 29.7)	66.1 (60.5, 72.5)	<0.001

mDFS, Median Disease Free Survival.

DFS, The time from diagnosis of breast cancer to the first recurrence.

mOS, Median Overall Survival.

OS, The time from diagnosis of breast cancer to dead.

Data are presented as median (months) (95%CI).

## 4 Discussion

To our knowledge, relative to other subtypes of breast cancer, TNBC was always associated with a higher aggressiveness and more risk of local recurrence and visceral metastasis (3–5). The highest risk of recurrence in TNBC patients was during the first 3 years after the disease diagnosis. After the first 3 years, the recurrence risk declined rapidly (11). Until recently, most TNBC studies have focused on overall recurred populations, and fewer studies have examined the timing of relapse. However, in our clinical practice, there was an aggressive subset of TNBC patients with marked chemoresistance, rapid metastatic spread, and poor survival. We defined it as the rapid-relapse TNBC (RR-TNBC). Learning more about the biological characteristics of RR-TNBC could help us to better identify these patients, improve patient outcomes, and optimize treatment regimens in the future. In our study, through the prognostic analysis of all 1584 TNBC patients and the characteristics of recurrence analysis in different recurred types of TNBC patients, no matter the median disease-free survival or the median overall survival, RR-TNBC patients have the worst prognosis, followed by SR-TNBC patients. This result was consistent with the observation of TNBC patients from the previous studies (17, 18). Besides that, RR-TNBC patients tended to have a higher T staging, N staging, TNM staging, and low expression of stromal tumor-infiltrating lymphocytes (sTIL). The recurrence characteristics were prone to appear as distant metastasis at the first relapse. The first metastatic site was apt to visceral metastasis and less likely to have chest wall or regional lymph node metastasis. We supposed that the worse prognosis of RR-TNBC patients may be due to: 1) the biological characteristics of TNBC; having no therapeutic target, high heterogeneity, and aggressive behavior: 2) just as we found in our study, through the comparative analysis between RR-TNBC and SR-TNBC groups, the population distribution and relapsed characteristics of RR-TNBC patients may also lead to an earlier recurrence and metastasis, and ultimately affect the prognosis: and 3) a lack of more detailed treatment information may also lead to a different prognosis.

In this study, we also included a series of non-RR-TNBC patients to assess the risk factors for relapse in RR-TNBC patients. We included many clinicopathological factors in predicting risk factors. Among these clinicopathological indicators, primary tumor size, lymph node metastatic status, tumor staging, lymph-vascular invasion or not, tumor grade, and Ki-67 status were important indicators that reflect the characteristics of triple-negative breast tumors. Related research has reported that TNBC patients with larger tumors, more lymph node metastases, later TNM staging,lymph-vascular invasion, higher tumor grade, and Ki-67 were more likely to have metastasis and had a shorter disease-free survival (19, 20). However, our study found that ≥pT3 staging and ≥pN1 staging were the independent risk factors for relapse in RR-TNBC patients. Patients with higher T staging and N staging did have a worse prognosis. The reason for this might be that TNBC patients with a higher T staging or N staging had greater tumor burdens. Therefore, compared with other clinicopathological indicators, ≥pT3 staging and ≥pN1 staging were important factors in rapid recurrence and metastasis.

Most oncologists agreed with that: except for the biological characteristics of the tumor itself, the tumor microenvironment also played an important role in the growth, invasion, and metastasis of tumor cells. Therefore, in our study, we also discussed whether some tumor microenvironment variables were associated with the rapid relapse in TNBC patients or not, such as sTILs, P53, CK5/6, and EGFR. Many studies have reported that TNBC with a high level of sTILs showed better short-term and long-term prognoses (21–23). The main reason for this was that the CD4+ T cells and CD8+ T cells (primary effector sTIL subtypes) had been linked to a better response to anti-tumor treatment in triple-negative breast cancer (24, 25). However, a small number of studies reported that TNBC patients with sTIL enrichment after NAC were at a higher risk of relapse (26). Through univariate and multivariate analysis in the training set, we found that the intermediate/high expression of sTILs was an independent protective factor for recurrence in RR-TNBC patients. Moreover, prognostic analysis of all TNBC patients confirmed that patients with a higher level of sTILs had a better prognosis, which was consistent with many previous reports. Although relevant studies have reported that the positive expression of CK5/6, EGFR, and P53 in TNBC patients had a worse prognosis (4, 27), our study did not find that CK5/6, EGFR, and P53 expression were associated with rapid relapse in TNBC patients. These results requires more studies and larger sample sizes to verify this conclusion.

Sociodemographic factor analysis by Daniel G. Stover et al. found that RR-TNBC patients were associated with Medicaid/indigent insurance, being single, of Black ethnicity, having a lower income, and a younger age at diagnosis (28, 29). Due to a lack of data, we did not focus on the analysis of many sociodemographic factors. Our clinicopathological factors analysis only found that postmenopausal status was an independent protective factor for recurrence in rrTNBC patients. There was no significant difference in overall survival between premenopausal and postmenopausal patients. Female patients of younger age (age ≤ 35 years) and family history were not significantly associated with the rapid relapse of TNBC patients. Moreover, we studied whether some special type of TNBC would increase the risk of rapid relapse or not. Few studies in this area were mainly because the number of these special types of TNBC patients was too small. Most research about the special type of TNBC patients focused on the prognosis. In our study, we also found that metaplastic breast cancer (MBC) patients had the worst prognosis. The five-year overall survival rate of MBC patients was about 60.0% (27/45), which was consistent with previous relevant reports (30–32). Besides that, we found that MBC was an independent risk factor for rapid relapse in TNBC patients. This may be associated with the biological characteristics of MBC. Considerating the low morbidity of MBC, and the small number of MBC patients in our study, more research is required to confirm this.

In recent years, Trastuzumab deruxtecan (T-DXd), a novel HER2-targeting therapy has been developed. Relevant research found that it can not only target HER2-positive tumor cells but also can effectively target tumor cells that express low levels of HER2 through the bystander effect to neighboring tumor cells heterogeneously expressing HER2 (33–35). Therefore, we explored the relationship between HER2 status and RR-TNBC in our study. Although the overexpression of HER2 was associated with shorter overall survival and a higher risk of disease recurrence. Through the univariate and multivariate analysis in our study, only HER2 (1+) status was an independent protective factor for recurrence in RR-TNBC patients. Moreover, survival analysis showed that patients with HER2 (1+) status had a better prognosis. Therefore, the relationship between HER2 status and RR-TNBC remains unclear and needs more research to confirm it. In our study, we also analyzed the relationship between different treatments and RR-TNBC patients. Findings indicated that breast surgery, chemotherapy, and radiotherapy reduce the risk of locoregional failure, recurrence, and breast cancer mortality in TNBC patients, especially patients with intermediate or high-recurring risk factors (36, 37). In our study, we did not find that different treatments were significantly associated with rapid relapse in TNBC patients.

Our study combined the results of univariate and multivariate analysis in the training set and established a predictive model for the rapid relapse in TNBC patients. Through the analysis of receiver operating characteristics (ROC) and the calibration curve in the verification set, we found that our predictive model revealed good discrimination with a C-index of 0.861 and good accuracy with a brier score of 0.095. These findings indicate that our predictive model is suitable for predicting the probability of rapid relapse in TNBC patients.

## 5 Limitations

Several limitations should be considered in our study: first, it included the heterogeneous TNBC patients and a retrospective study from a single center. Second, it lacked more sociodemographic, genomic data, and detailed treatment information may weaken the accuracy of prediction. For example, the number of chemotherapy regimen cycles, the dose of chemotherapy and radiotherapy, the completion rate of chemoradiotherapy, and whether chemotherapy and radiotherapy were delayed or not. Finally, the generalizability of the predictive model needs to be externally validated with an independent population before it can be applied to clinical practice. Therefore, we still need a larger sample size and longer follow-up time to analyze the biological characteristics of RR-TNBC patients in the future.

## 6 Conclusion

In this single-center study, by studying the characteristics of recurrence, predictors for relapse, and prognosis in RR-TNBC patients, we verified that TNBC was a highly heterogeneous disease. The different relapse types of TNBC patients had unique biological behaviors and prognoses, especially for RR-TNBC patients. Addressing the limitations of the present study, we will continue to expand our sample sizes and collect more data from different breast cancer treatment centers in the future, to build a better predictive model through internal and external validation methods. In addition, integrating more sociodemographic, clinicopathological, and genomic features in the future will help us excavate more internal and external biological characteristics for RR-TNBC patients. Following on from the progress we have made in immunotherapy and antibody–drug conjugates therapy (35, 38–40), this exploratory and comprehensive research on RR-TNBC patients will help us to find more potential therapeutic targets and optimize treatment regimens, benefitting TNBC patients through individualized treatment.

## Data availability statement

The original contributions presented in the study are included in the article/supplementary material. Further inquiries can be directed to the corresponding author.

## Ethics statement

The studies involving human participants were reviewed and approved by Medical Ethics Committee of Tianjin Medical University Cancer Institute and Hospital. The patients/participants provided their written informed consent to participate in this study.

## Author contributions

S-LC and JZ contributed to the conception and design of the study. S-LC and SZ developed the methodology. X-QL, H-DC, XF, TM, YL, Y-QL, C-YL, SZ, X-GC, and X-JG took part in the data acquisition, analysis, and interpretation. S-LC, J-JL, Y-XL, S-HY, and XL wrote, reviewed, and/or revised the manuscript. JZ supervised the study. All authors contributed to the article and approved the submitted version.

## Glossary

**Table:**

BC	breast cancer
TNBC	triple-negative breast cancer
RR-TNBC	rapid relapse triple-negative breast cancer
SR-TNBC	slow relapse triple-negative breast cancer
NR-TNBC	no relapse triple-negative breast cancer
Non-RR-TNBC	non-rapid relapse triple-negative breast cancer
ER	estrogen receptor
PR	progesterone receptor
HER2	human epidermal growth factor receptor 2
CK5/6	cytokeratin 5/6
EGFR	epidermal growth factor receptor
sTILs	stromal tumor-infiltrating lymphocytes
mOS	Median overall survival
mDFS	Median disease-free survival
DCIS	ductal carcinoma *in situ;* LCIS, lobular carcinoma in situ
MBC	metaplastic breast cancer
HR	hormone receptor
HR+	HR-positive
HR-	HR-negative
HER2+	HER2 positive
HER2-	HER2 negative
IHC	immunohistochemistry
FISH	fluorescence *in situ* hybridization
ROC	receiver operating characteristic
AUC	area under the curve
